# Cerebrovascular Reactivity Measures Are Associated With Post-traumatic Headache Severity in Chronic TBI; A Retrospective Analysis

**DOI:** 10.3389/fphys.2021.649901

**Published:** 2021-05-13

**Authors:** Franck Amyot, Cillian E. Lynch, John Ollinger, J. Kent Werner, E. Silverman, Carol Moore, Cora Davis, L. Christine Turtzo, Ramon Diaz-Arrastia, Kimbra Kenney

**Affiliations:** ^1^National Intrepid Center of Excellence, Walter Reed National Military Medical Center, Bethesda, MD, United States; ^2^Department of Neurology, Perelman School of Medicine, University of Pennsylvania, Philadelphia, PA, United States; ^3^Department of Neurology, Uniformed Services University of the Health Sciences, Bethesda, MD, United States; ^4^National Institutes of Neurological Disorders and Stroke, National Institutes of Health, Bethesda, MD, United States

**Keywords:** cerebrovascular reactivity, migraine, chronic, traumatic brain injury, post-traumatic headache

## Abstract

**Objective:**

To characterize the relationship between persistent post-traumatic headache (pPTH) and traumatic cerebrovascular injury (TCVI) in chronic traumatic brain injury (TBI). Cerebrovascular reactivity (CVR), a measure of the cerebral microvasculature and endothelial cell function, is altered both in individuals with chronic TBI and migraine headache disorder (Amyot et al., 2017; Lee et al., 2019b). The pathophysiologies of pPTH and migraine are believed to be associated with chronic microvascular dysfunction. We therefore hypothesize that TCVI may contribute to the underlying migraine-like mechanism(s) of pPTH.

**Materials and Methods:**

22 moderate/severe TBI participants in the chronic stage (>6 months) underwent anatomic and functional magnetic resonance imaging (fMRI) scanning with hypercapnia gas challenge to measure CVR as well as the change in CVR (ΔCVR) after single-dose treatment of a specific phosphodiesterase-5 (PDE-5) inhibitor, sildenafil, which potentiates vasodilation in response to hypercapnia in impaired endothelium, as part of a Phase2a RCT of sildenafil in chronic TBI (NCT01762475). CVR and ΔCVR measures of each participant were compared with the individual’s pPTH severity measured by the headache impact test-6 (HIT-6) survey.

**Results:**

There was a moderate correlation between HIT-6 and both CVR and ΔCVR scores [Spearman’s correlation = –0.50 (*p* = 0.018) and = 0.46 (*p* = 0.03), respectively], indicating that a higher headache burden is associated with decreased endothelial function in our chronic TBI population.

**Conclusion:**

There is a correlation between PTH and CVR in chronic moderate-severe TBI. This relationship suggests that chronic TCVI may underlie the pathobiology of pPTH. Further, our results suggest that novel treatment strategies that target endothelial function and vascular health may be beneficial in refractory pPTH.

## Introduction

There is a high prevalence of persistent post-traumatic headache (pPTH), a newly developing or worsening headache disorder following a TBI that persists at least 3 months, among chronic traumatic brain injury (TBI) survivors (Faux and Sheedy, 2008), with 47–95% reporting frequent disabling headaches that are majority migraine-like (Headache Classification Committee of the International Headache Society [IHS], 2013; Defrin, 2014). A latterly published study by Metti et al. (2020) has reported PTH characteristics, frequency, severity and outcomes of a cohort of recently deployed soldiers (*n* = 1,587) enrolled in the Warrior Strong epidemiological study at Forts Bragg and Carson. Headaches were classified based on a detailed phenotypic questionnaire that incorporates International Classification of Headache Disorders, version 3 (ICHD-3) criteria (Headache Classification Committee of the International Headache Society [IHS], 2018) for the primary headache disorders most commonly reported in PTH. The most common PTH phenotype was migraine headache, but, more importantly, active duty service members with PTH had more phenotypically complex headaches than those with headache disorders that were not associated with TBI (Metti et al., 2020). Specifically, in this large cohort of active duty service members with and without a history of a recent mTBI, PTH was associated with more features including severity, allodynia, visual/sensory aura, and daily or continuous occurrence compared to headaches presumed unrelated to TBI. While long term PTH outcomes and its impact on military fitness, readiness and retention have not been fully characterized, migraine remains among the most common diagnoses listed in military medical discharges (Accession Medical Standards Analysis and Research Activity, 2019).

However, the mechanisms underlying post-traumatic headache (PTH) are not yet well understood. Possible causal mechanisms include axonal and/or vascular injury, neuroinflammation, and alteration in cerebral metabolism. Likewise, the pathophysiology of migraine headache disorder has not yet been definitively established, but current leading hypotheses focus on vascular etiologies (Mason and Russo, 2018). Traumatic cerebrovascular injury (TCVI) is a nearly universal endophenotype of TBI and may be partially responsible for TBI-related chronic disability. We hypothesized that pPTH may result from chronic traumatic endothelial cell and microvasculature dysfunction.

Cerebrovascular reactivity (CVR) is the ability of the cerebral vasculature to constrict or dilate under varying physiological conditions and is believed to reflect the responsiveness of the cerebral microvasculature networks. Well-established non-invasive imaging methods are available that accurately measure CVR. One method includes functional magnetic resonance imaging (fMRI) in conjunction with an exogenous stimulus such as hypercapnia or breath holding (Kassner and Roberts, 2004; Mutch et al., 2016a, b, 2018; Shafi et al., 2020). By imaging the changes in MRI-Blood Oxygen Level Dependent (MRI-BOLD) signaling during a hypercapnia challenge, one can generate whole brain CVR maps (Lu et al., 2014). Altered CVR measures have also been implicated in migraine pathophysiology, both globally and focally in areas of white matter hyperintensities (WMH) on fluid-attenuated inversion recovery (FLAIR) imaging (Lee et al., 2019b). Recently, we have demonstrated multiple focal CVR deficits in a chronic moderate-severe TBI population (Amyot et al., 2017) and that focal CVR deficits are partly responsive to single dose phosphodiesterase-5 (PDE5) inhibitors (Kenney et al., 2018; Lee et al., 2019b).

In this study we investigated the relationship between PTH severity and CVR deficit in chronic TBI subjects as assessed by the HIT-6 survey and MRI-BOLD with hypercapnia challenge, respectively. In addition, we aimed to determine whether impaired endothelial cell function and consequently decreased permissive action of intrinsic nitric oxide (NO) production on CVR may underlie the pathobiology of PTH and whether CVR and ΔCVR (the change in CVR measurement from baseline after a single dose of a PDE-5 inhibitor during MRI-BOLD with hypercapnia challenge) may be functional imaging biomarkers of PTH.

## Methods

### Study Population

We included a convenience sample of 22 TBI participants between 18 and 55 years of age who were consented and enrolled under an IRB-approved clinical trial (NCT01762475) in our analysis. We also enrolled 15 age, sex and education matched controls (HC) without a history of TBI (Kenney et al., 2018). Inclusion criteria for TBI subjects included chronic moderate or severe (by VA-DoD criteria) TBI between 6 months and 10 years since injury. Exclusion criteria included penetrating TBI, pre-existing disabling neurologic or psychiatric disorder, pregnancy, unstable pulmonary or vascular disorder, or contraindications to taking sildenafil.

### CVR Scan

MRI was performed on a fully integrated 3 Tesla MRI/PET. T1 Magnetization Prepared Rapid Gradient Echo (MPRAGE) and MRI-BOLD sequences were acquired on each subject. The MRI-BOLD sequence parameters were TR/TE = 2,000/25 ms, flip angle = 80°, field of view = 220 9 220 mm^1^, matrix = 64 9 64, 36 slices, thickness = 3.6 mm, no gap between slices, 210 volumes. Each participant underwent MRI-BOLD with hypercapnia challenge before and after a single dose of sildenafil (Kenney et al., 2018). Hypercapnia challenge was induced via a block design of gas inhalation through a face mask with alternating flow every minute between room air and 5% carbon dioxide (CO_2_)-room air admixture for 7 min total while the MRI-BOLD images were acquired. End-tidal CO_2_ (EtCO_2_) was measured continuously during the hypercapnia challenge (Amyot et al., 2017). To measure the change in CO_2_-induced CVR from baseline following phosphodiesterase-5 (PDE-5) inhibition (ΔCVR), a single 50 mg oral dose of sildenafil was administered to each participant, and a second hypercapnia challenge was performed 1 h later as previously described (Kenney et al., 2018). Images were spatially realigned and re-sliced to correct for head motion with SPM (realign and re-slice toolbox). We monitored six parameters (x, y, z translation and pitch, yaw and roll) to assure correct co-registration. Scans from 2 of the 24 TBI subjects initially enrolled in the study for whom either head motion was not correctable, or BOLD fMRI was not available due to technical issues were not analyzed.

### CVR and ΔCVR Measurements and Maps

**FIGURE 1:**
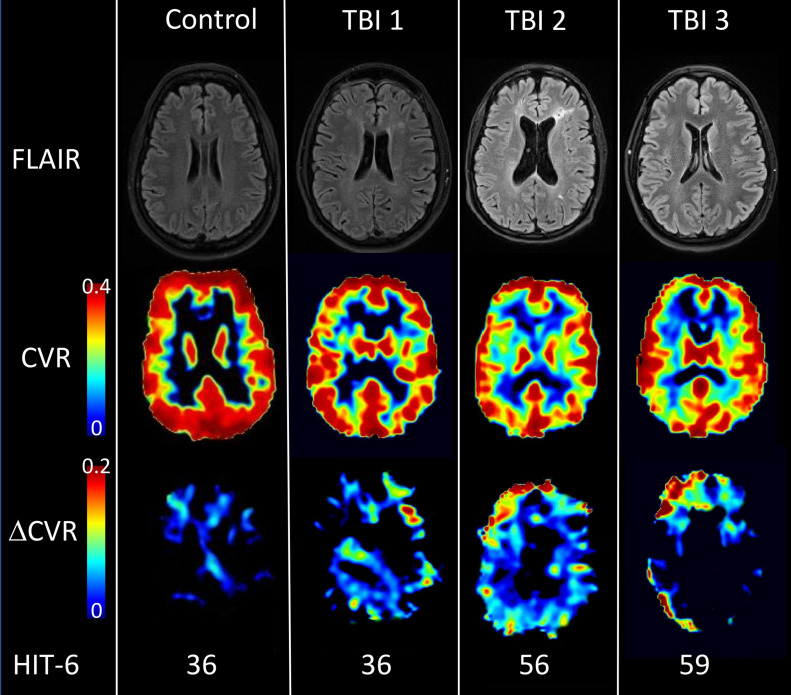
Structural imaging, CVR and ΔCVR maps for representative participants. Brain images of one healthy control (Female, 49 yo) and three TBI acquired with FLAIR (first row) and fMRI BOLD during hypercapnia challenge (2nd and 3rd row). The second row displays CVR maps at baseline and the third row the difference between two hypercapnia challenges due to oral ingestion of 50 mg sildenafil (ΔCVR). The control subject displayed high CVR values in gray matter and low CVR values in white matter, with a mean CVR value of 0.227%BOLD/mmHg in the whole brain. For the control, sildenafil didn’t potentiate CVR (ΔCVR image) with ΔCVR = –0.005%BOLD/mmHg. However, TBI subjects (with or without FLAIR hyperintensity) exhibited focal CVR deficits which are generally potentiated by 50mg sildenafil. Each subject was characterized by their HIT-6 score (36 for TBI#1, 56 for TBI#2 and 59 for TBI#3); CVR value (0.190, 0.183, and 0.152%BOLD/mmHg) and ΔCVR values (0.010, 0.183, and 0.023%BOLD/mmHg).

For each participant, voxel-by-voxel CVR maps were calculated based on a linear regression between BOLD signal time courses and EtCO_2_ time regression using Statistical Parametric Mapping (Figure 1). Whole brain CVR value was calculated as the average of the 3D CVR voxel map for each participant. Whole brain ΔCVR and voxel-level ΔCVR maps were calculated as the difference between CVR measurements at baseline and CVR measurements 30–60 min following a single-dose sildenafil. Following the single-dose sildenafil study, the 22 symptomatic TBI subjects continued into an 8 week single daily oral dose sildenafil trial to assess the effect of chronic CVR facilitation on ΔCVR by comparison of a single 8 week MRI BOLD hypercapnic challenge CVR value with the initial pre-sildenafil baseline MRI BOLD hypercapnic challenge CVR pre-trial values 8 weeks prior. Neurological assessment, including pPTH score, was recorded for all participants following 8 week treatment.

### HIT-6

Participant headache severity was assessed by a validated headache survey, the Headache Impact Test-6 (HIT-6), which surveys headache severity and burden over the prior 4 weeks. It is a 6-item survey that asks individuals to rate pain severity and headache effects on daily activities, ability to work, mood and attention (see text footnote 1). Each item is rated qualitatively at 5 levels (never, rarely, sometimes, very often, always) and scored on a corresponding range from 6 to 13. A sum score of ≤49 has little or no headache impact, 50–55 some, 56–59 substantial and ≥60 severe impact. The HIT-6 was administered just prior to imaging at each study visit. A clinically significant difference has been determined to be an increase or decrease of ≥6 points on the HIT-6 score (Yang et al., 2011). Of all subjects included in this report, only one study participant included in this analysis, a healthy control, had a Prior Medical History (PMH) of migraine, with one TBI participant having a PMH of non-migrainous headache.

### CVR, ΔCVR, and HIT-6 Correlation

Prior to Spearman Correlation analysis of global CVR values and HIT-6 score, a Principal Component Analysis (PCA) was run by the statistical software package R “procomp” function in order to reduce the number of variables and develop a better understanding of the driving forces that generated the data. Each variable was scaled to its own standard deviation to form the covariance matrix (Supplementary Figure 1). The eigenvectors form the direction of each component. The eigenvalues give the explained variance or the importance of each component. Subsequent to PCA, three separate Spearman rank correlations (CVR vs. HIT-6, ΔCVR vs. HIT-6, and CVR vs. ΔCVR) were carried out on ranked data.

## Results

### Demographics

Twenty-two chronic moderate-severe TBI and 15 HCs were consented and enrolled in the study and 22 had adequate image resolution for analysis (Table 1). One healthy control reported a prior history of migraines and one TBI participant reported a prior history of non-migrainous headache disorder. Mean HIT-6 scores at baseline were 50 ± 11 and 42 ± 8 (*p* = 0.011) for TBI and HC subjects, respectively. No participants self-reported increased headache severity either following single dose sildenafil administration or BOLD MRI with hypercapnic challenge or reported association of headache with daily sildenafil intake over the course of the 8 week trial.

**TABLE 1:** Demographic and TBI characteristics.

	TBI (*n* = 22)	HC (*n* = 15)	*p*-value
Age [years; Mean ± *SD* (IQR)]	38 ± 11 (29–49)	38 ± 8 (34–42)	0.49
Sex (% male)	80%	80%	0.47
Education [years; Mean ± *SD* (IQR)]	15 ± 3.0 (13–16)	17 ± 3 (15–20)	0.018
Fazekas scale (Median, IQR)	1 (0, 1)	0 (0, 1)	0.011
Road accident (%)	70%	–	–
Loss of consciousness (%)	100%	–	–
Intensive care unit admission (%)	100%	–	–
Abnormal neuroimaging (%)	95%	–	–
Time from injury [months; Median (IQR)]	44 (33–128)	–	–
HIT score mean ± *SD* (IQR)	50 ± 11 (IQR, 40–58)	42 ± 8 (IQR, 39–56)	0.011

### CVR Map

Figure 1 shows FLAIR, CVR, and ΔCVR images from one HC and three TBI subjects with HIT-6 scores of 36, 36, 56, and 59; whole brain CVR values of 0.227%BOLD/mmHg for the HC, and 0.196, 0.183, and 0.150%BOLD/mmHg for the three TBI subjects; whole brain ΔCVR measures of -0.005, 0.01, 0.023, and 0.020%BOLD/mmHg, respectively. WMHs are common in our TBI population (Figure 1, TBI#1 and TBI#3) and often, but variably, co-localize with focal CVR deficits.

### CVR Quantification

The CVR values of individual participants were assessed by averaging all CVR voxels across the whole brain. For the 22 chronic TBI, mean whole brain CVR value was 0.186 ± 0.025%BOLD/mmHg. After 50mg sildenafil administration, mean whole brain CVR significantly increased to a value of 0.207 ± 0.023%BOLD/mmHg (Paired *t*-test, *p* < 0.0001) with a mean ΔCVR measure of 0.021 ± 0.012%BOLD/mmHg. Mean CVR in controls was 0.223 ± 0.010%BOLD/mmHg with no significant mean ΔCVR (Supplementary Figure 2). Prior to single-dose sildenafil, the average and standard deviation end-tidal CO_2_ across both healthy control and TBI patients in this study was 39.9 ± 1.6 mmHg at baseline prior to hypercapnic challenge, with a rise to 49 ± 1.2 mmHg upon hypercapnic challenge. Following single-dose sildenafil, the average and standard deviation ETCO_2_ across both healthy control and TBI subjects was 40.1 ± 1.5 mmHg at baseline, rising to a post sildenafil ΔCO_2_ of 48.6 ± 1.8 mmHg during hypercapnia. There was no difference between mean baseline ETCO_2_ or ΔETCO_2_ between control and TBI groups (HC: at baseline, ETCO_2_ = 40.3 ± 1.9. Under 5% CO_2_, EtCO_2_ = 50.1 ± 1.5 mmHg; TBI at baseline, EtCO_2_ = 39.7 ± 2.2. Under 5% CO_2_, EtCO_2_ = 49.9 ± 1.9 mmHg).

### Correlation Between HIT-6 and CVR

**FIGURE 2:**
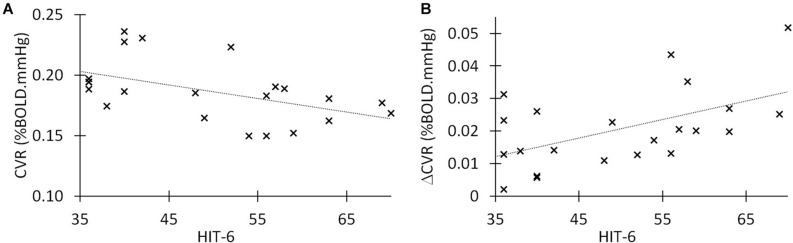
Correlations between HIT-6, CVR, and ΔCVR in chronic TBI. **(A)** Linear correlation between post-traumatic headache (HIT-6 score) and cerebrovascular reactivity in 22 symptomatic TBI patients in chronic stage. Spearman r = −0.50 (p = 0.018). **(B)** Linear correlation between delta CVR (potentiation of CVR after 50 mg of sildenafil) and HIT-6 score in our group of 22 moderate/severe TBI. The Spearman’s r = 0.46 (p = 0.03).

For each TBI participant (*n* = 22), we associated CVR and ΔCVR values with the headache score (HIT-6). Figure 2A shows the correlation between CVR and HIT-6 across subjects. We found a moderate but negative correlation between HIT-6 and CVR, indicating that a higher HIT-6 score is associated with a lower whole brain CVR measure. Figure 2B shows the relationship between ΔCVR and headache severity score, also demonstrating a moderate *positive* correlation, meaning a greater ΔCVR measure is associated with a lower HIT-6 score (Spearman Correlation = −0.48, *p* = 0.016). Conversely, CVR was negatively correlated with HIT-6 score (Spearman correlation = −0.50, *p* = 0.018). We performed a scaling Principal Component Analysis (PCA) between CVR, ΔCVR, and HIT-6, and found that 85% of all dataset variabilities lie along two principal components, showing a high correlation among these variables. There was a trend in the increase of CVR from baseline values following 8 week daily dose sildenafil, however, no significant change was seen between pre- and post-sildenafil HIT-6 scores. In addition, no participant reported any increased headache severity or occurrence after sildenafil self-administration.

## Discussion

In this small cohort of chronic TBI patients with pleomorphic TBI-related imaging abnormalities (WMH, multifocal encephalomalacia, diffuse TCVI), we found a relationship between CVR and pPTH, similar to that recently reported between CVR and WMH burden in non-TBI-related migraine headache disorder (Lee et al., 2019b). With further study, this association may provide valuable insights into the pathobiology of pPTH in chronic TBI. The moderately negative correlation between HIT-6 and whole brain CVR indicates that a higher headache burden is associated with a higher burden of TCVI. We also found that the improvement in endothelial function after sildenafil administration (higher ΔCVR measure), observed only in TBI participants, is also associated with a lower headache burden, perhaps indicating that pPTH phenotype is determined not just by the degree of cerebral endothelial dysfunction, but also by that of micro-vasculopathy and mural cell health in general. Further, CVR and ΔCVR measures may be functional imaging biomarkers of chronic vascular dysfunction and should be further explored in patients with pPTH. Finally, among TBI subjects, akin to that recently reported in chronic migraineurs, FLAIR WMHs frequently co-localize with focal CVR deficits (Amyot et al., 2017; Haber et al., 2018).

Because this was a *post hoc* analysis of the possible physiological relationship between microvascular injury and dysfunction and severity of pPTH in a cohort previously assessed for CVR impairment alone (Amyot et al., 2017; Haber et al., 2018), we do not report the effect of single-dose sildenafil treatment on pPTH severity. The HIT-6 is a measure of headache burden over the prior 4 weeks and not a measure of headache severity at the time CVR testing was performed. No participants in the study reported increased headache pain after single dose sildenafil administration for ΔCVR measurements. Since acute migraine-like headache is a well-established, common (∼10%) side effect of sildenafil administration, it is unlikely that a transient and indirect pharmacological rescue of CVR deficit would acutely ameliorate headache intensity, as reflected by HIT-6 score, alongside potentiated ΔCVR values. In fact, acute sildenafil administration is known to trigger headache in migraineurs at doses sub-optimal for middle cerebral arterial vasodilation (Kruuse et al., 2003), and it induces cluster headaches in healthy individuals with a history of headache bouts (Lin et al., 2014). This triggering is likely due to the cephalic intradural vasoactive effects of sildenafil (Christensen et al., 2019; Christensen et al., 2021) that are distinct from the intra-cerebral vasodilatory actions causally linked to diminished CVR and downstream WMH (Stanimirovic and Friedman, 2012; Sam et al., 2016; Mascalchi et al., 2017) in the context of migraine (Mason and Russo, 2018; Lee et al., 2019a, b) and pPTH (O’Neil et al., 2013; Clark et al., 2017; Haber et al., 2018).

Although we did not record the HIT-6 score immediately following single-dose sildenafil administration, no participants reported headache as an acute adverse event. Further, the same cohort of moderate-to-severe TBI patients were assessed for potentiation of ΔCVR immediately prior to, and following 8 weeks of a single daily dose administration of sildenafil, and examined for a battery of neurological and self-reported quality of life outcomes following the 8 week trial (Kenney et al., 2018). Of these 22 symptomatic TBI patients, despite a trend in increase of ΔCVR from baseline values, change in headache phenotype with daily sildenafil treatment was not noted, and 11 patients showed no change in HIT-6 score from baseline pre-trial values. Headache score increased by 6 or more points on the HIT-6 for four patients, and decreased in seven (Kenney et al., 2018). These inconsistent and non-significant HIT-6 scores following chronic sildenafil treatment may support the hypothesis that pharmacologic facilitation of CVR, especially in chronic TBI dependent TCVI, may be ineffective due to the late introduction date when irreversible CVR-related WMHs have occurred (Amyot et al., 2017), similar with disease progression in a cohort of migraineurs (Lee et al., 2019b), and with aging in healthy subjects in general (Sam et al., 2016). Although it has been reported that older adult patients prescribed statins, which is known to experimentally restore CVR (Tong et al., 2012; Tong and Hamel, 2015), exhibit WMH burden similar to age-matched control subject (Soljanlahti et al., 2007), indicating preservation of vascular health, and presumably CVR, may slow white matter lesions, there is, to our knowledge no other study, which suggests that pharmacotherapeutic targeting of CVR impairment in chronic TBI patients or migraineurs may be protective against CVR related pPTH or migraine phenotype, respectively. Further research is needed to address these important questions.

As is often unavoidable with human studies, the enrolled cohort of TBI patients were heterogenous in age and duration of disease. This may have given rise to a more skewed distribution of ΔCVR correlations in our analysis than would be seen had the patients been more similar demographically. However, it is interesting to note that a recent study by Lee and colleagues (Lee et al., 2019a) demonstrated the age of onset of migraine correlates with worse CVR reduction, with younger patients displaying greatest CVR impairment, and a paradoxical negative effect of duration of disease on this correlation, indicating that younger individuals may be particularly vulnerable to CVR associated migraine (Lee et al., 2019a). Our TBI patients were inhomogeneous in both age and time post-injury, and this may have masked an increased susceptibility of the younger arm of our patients to a CVR-related pPTH (Lee et al., 2019a). Indeed, it is well accepted that mTBI at younger age results in slower resolution of post-concussive symptom (PCS) burden, including PTH (Alosco et al., 2017; McCrory et al., 2017; Harmon et al., 2019), and engaging in early physical activity, which is known to be positively correlated with CVR in healthy individuals (Barnes et al., 2013; Hwang et al., 2018), has been shown to reduce PCS duration and likelihood of PTH following pediatric sports related concussion (SRC) (Wilson et al., 2020).

This study has several limitations, including the overall small size, predominant male sex and inclusion of moderate and severe TBI survivors only, limiting its generalizability to the chronic TBI population which is predominantly mild TBI (mTBI). In addition, the majority of our TBI cohort (15 of 27 patients) displayed pleomorphic imaging abnormalities, including diffuse WMH burden and multifocal parenchymal lesions (Amyot et al., 2017; Haber et al., 2018). A caveat should be noted in interpreting the contribution of TCVI related CVR deficit to a possible WMH-related pPTH phenotype; it is impossible to determine what proportion of WMH burden is attributable to the initial primary injury vs. the downstream secondary insult of protracted ischemic lesions. It has been reported that CBF in the cingulate cortex of veterans with a history of TBI correlates with compromised white matter integrity in those subjects furthest removed from their injury (Clark et al., 2017), indicating the lesion-associated diffuse white matter and microvascular injury in chronic head trauma may further perturb white matter form and function long after the primary mechanical injury (Clark et al., 2017). Regardless, this is an important point to consider with respect to comparison of our data with that available for episodic migraine, in which no external trauma is pathophysiologically associated with disease incidence.

The underlying pathophysiology of PTH remains unknown, hindering the development of effective therapies. Both migraine and TBI are associated with cerebral microvascular dysfunction, in particular with cerebral endothelial cell dysfunction. Multiple non-invasive modalities have been shown to reliably measure CVR after TBI and we have established that CVR is a biomarker of the TCVI endophenotype in chronic TBI. CVR is frequently decreased in migraine headache disorder. From this *post hoc* analysis of a cohort of chronic moderate-severe TBI survivors, we found that decreased CVR measures correlated with increased PTH disability, as measured with the HIT-6 headache disability scale, supporting a potential pathomechanistic relationship between TCVI and PTH. If these findings are confirmed in a larger cohort including pPTH sufferers after mTBI, it may support the investigation of therapies targeting endothelial function in pPTH patients. Such an approach would allow for precision medicine, endophenotype-specific, targeted treatments for refractory and debilitating pPTH. Further, CVR measures may prove to be prognostic as well as predictive and pharmacodynamic imaging biomarkers of pPTH that could be used clinically in TBI patients and experimentally in randomized clinical trials of therapies for this frequently disabling and recalcitrant chronic headache disorder.

## Disclosure

The identification of specific products or scientific instrumentation is considered an integral part of the scientific endeavor and does not constitute endorsement or implied endorsement on the part of the author, DoD, or any component agency. The views expressed in this manuscript are those of the author and do not reflect the official policy of the Department of Army/Navy/Air Force, Department of Defense, or U.S.Government.

## Data Availability Statement

The original contributions presented in the study are included in the article/Supplementary Material, further inquiries can be directed to the corresponding author/s.

## Ethics Statement

The studies involving human participants were reviewed and approved by the CNS IRB, NIH, Bethesda, MD. The patients/participants provided their written informed consent to participate in the study.

## Author Contributions

FA, KK, and RD-A conceived and implemented the study, collected and analyzed the data, and prepared the manuscript for publication. CL prepared and reviewed the manuscript for publication. CM and ES enrolled study subjects and collected the data. JO, JW, CD, and LT reviewed the manuscript for publication.

## Conflict of Interest

The authors declare that the research was conducted in the absence of any commercial or financial relationships that could be construed as a potential conflict of interest.

## Footnotes

1 https://pubmed.ncbi.nlm.nih.gov/20819842

## Supplementary Material

Supplementary Figure 1 Principal component analysis of relationship between CVR, ΔCVR, and HIT-6 score. Biplot of three vectors (in red) representing the contribution of CVR, Delta CVR and HIT6 to the two first components. Each black point represents the score of an observation (data issue from a subject in the PC space). The angle between two vectors represents the correlation between the variables. For example, the small angle between CVR and HIT-6 indicates a positive correlation. Contrary, an angle close to the 180° delineation indicates a negative correlation (as is the case for ΔCVR and HIT-6). An angle of 90° shows no correlation. The two first components contain 85% of the explained variance showing the data is mainly driven by the two first components.

Supplementary Figure 2 Comparison of group mean CVR and ΔCVR values. Bar graph of mean Healthy Control (blue) and TBI (red) group hypercapnic challenge evoked CVR at baseline and after single oral dose sildenafil. Healthy Controls demonstrated a mean global CVR in response to hypercapnic challenge pre-sildenafil administration of 0.224 ± 0.014 %BOLD/mHg versus 0.218 ± 0.016 %BOLD/mmHg post-sildenafil (Unpaired *t*-test, *p* > 0.05). The mean global CVR value of TBI subjects post-sildenafil was significantly increased to a value of 0.207 ± 0.023 %BOLD/mmHg, as compared to the baseline pre-sildenafil group mean CVR value of 0.187 ± 0.025 %BOLD/mmHg (Unpaired *t*-test, *p* < 0.001). Healthy Control (HC; Blue, *n* = 15), TBI (Red, *n* = 22), CVR values represent Mean ± *SD*.
