# Supplementary figures and images for: Cerebrovascular Reactivity Measures Are Associated With Post-traumatic Headache Severity in Chronic TBI; A Retrospective Analysis
Source: Front Physiol. 2021 May 13;12:649901. doi: 10.3389/fphys.2021.649901 (PMC8155500; doi:10.3389/fphys.2021.649901)

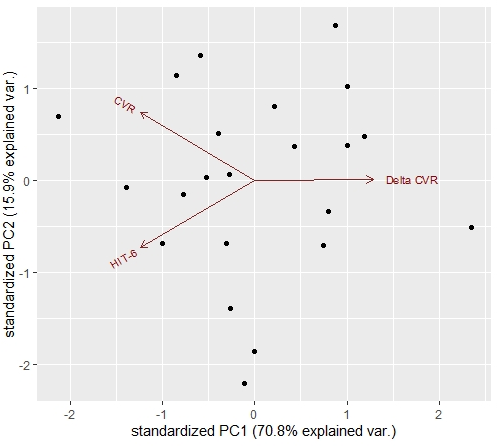

Supplement: Supplementary Figure 1 — Principal component analysis of relationship between CVR, ΔCVR, and HIT-6 score. Biplot of three vectors (in red) representing the contribution of CVR, Delta CVR and HIT6 to the two first components. Each black point represents the score of an observation (data issue from a subject in the PC space). The angle between two vectors represents the correlation between the variables. For example, the small angle between CVR and HIT-6 indicates a positive correlation. Contrary, an angle close to the 180° delineation indicates a negative correlation (as is the case for ΔCVR and HIT-6). An angle of 90° shows no correlation. The two first components contain 85% of the explained variance showing the data is mainly driven by the two first components. [file Image_1.tif]

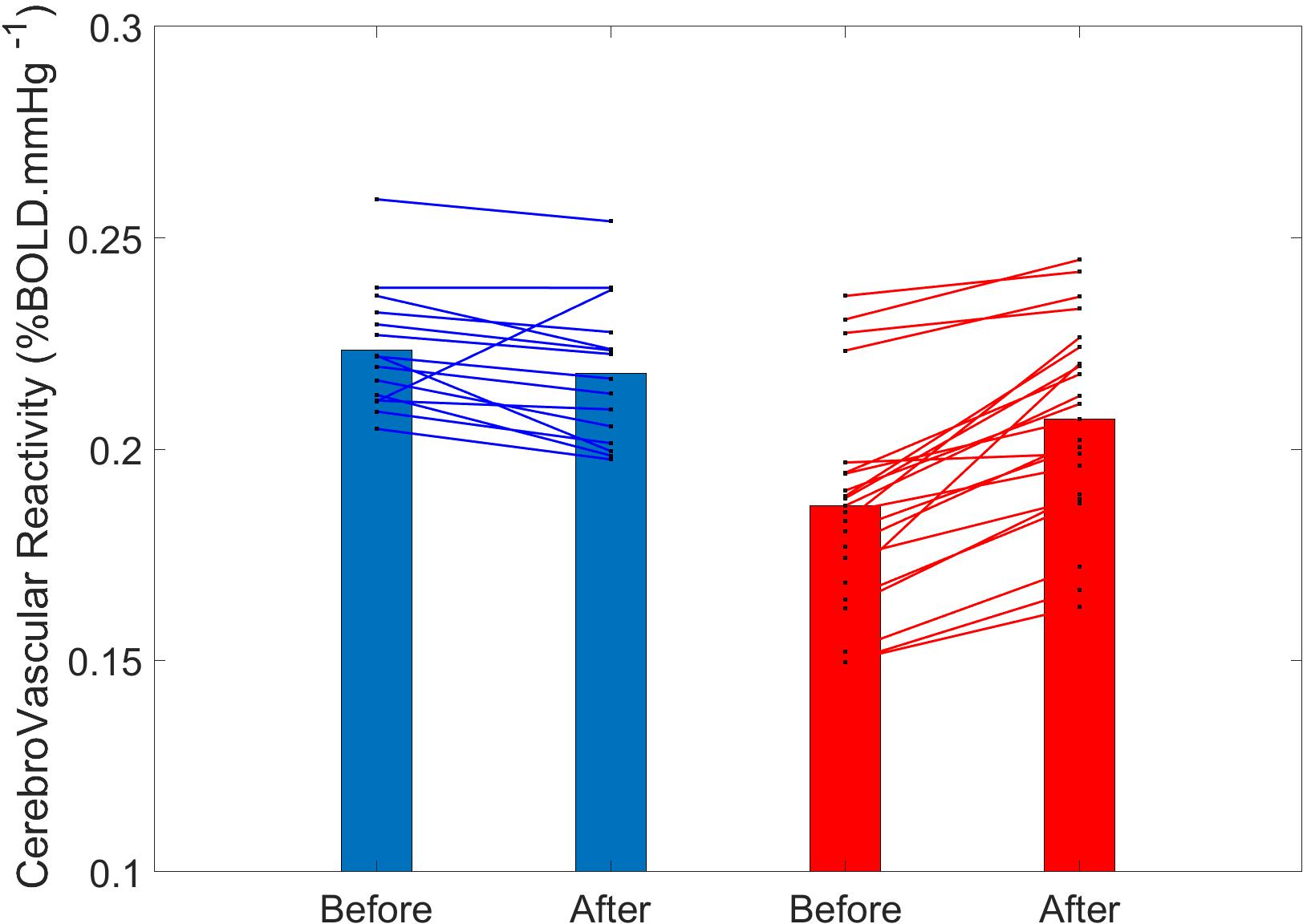

Supplement: Supplementary Figure 2 — Comparison of group mean CVR and ΔCVR values. Bar graph of mean Healthy Control (blue) and TBI (red) group hypercapnic challenge evoked CVR at baseline and after single oral dose sildenafil. Healthy Controls demonstrated a mean global CVR in response to hypercapnic challenge pre-sildenafil administration of 0.224 ± 0.014 %BOLD/mHg versus 0.218 ± 0.016 %BOLD/mmHg post-sildenafil (Unpaired t-test, p > 0.05). The mean global CVR value of TBI subjects post-sildenafil was significantly increased to a value of 0.207 ± 0.023 %BOLD/mmHg, as compared to the baseline pre-sildenafil group mean CVR value of 0.187 ± 0.025 %BOLD/mmHg (Unpaired t-test, p < 0.001). Healthy Control (HC; Blue, n = 15), TBI (Red, n = 22), CVR values represent Mean ± SD. [file Image_2.tif]
